# Socioeconomic Status, the Countries’ Socioeconomic Development and Mental Health: Observational Evidence for Persons with Spinal Cord Injury from 22 Countries

**DOI:** 10.3389/ijph.2022.1604673

**Published:** 2022-11-30

**Authors:** Christine Fekete, Hannah Tough, Annelie Schedin Leiulfsrud, Karin Postma, Andrea Bökel, Piotr Tederko, Jan D. Reinhardt

**Affiliations:** ^1^ Swiss Paraplegic Research, Nottwil, Switzerland; ^2^ Department of Health Sciences and Medicine, University of Lucerne, Lucerne, Switzerland; ^3^ Department of Neuromedicine and Movement Science, Faculty of Medicine and Health Sciences, Norwegian University of Science and Technology, Trondheim, Norway; ^4^ Department of Spinal Cord Injuries, Clinic of Physical Medicine and Rehabilitation St Olav’s University Hospital, Trondheim, Norway; ^5^ Erasmus Medical Center, Rotterdam, Netherlands; ^6^ Rijndam Rehabilitation, Rotterdam, Netherlands; ^7^ Department of Rehabilitation Medicine, Hannover Medical School, Hannover, Germany; ^8^ Department of Rehabilitation, Medical University of Warsaw, Warsaw, Poland; ^9^ Institute for Disaster Management and Reconstruction of Sichuan University and Hong Kong Polytechnic University, Sichuan University, Chengdu, China; ^10^ Jiangsu Province Hospital/Nanjing Medical University First Affiliated Hospital, Nanjing, China; ^11^ XD Group Hospital, Xi’an, China

**Keywords:** mental health, spinal cord injury, InSCI community survey, socioeconomic status, socioeconomic development, social inequalities, physical impairments

## Abstract

**Objectives:** Evidence on social inequalities in mental health of persons with physical impairments is limited. We therefore investigate associations of individual-level socioeconomic status (SES) and the country-level socioeconomic development (SED) with mental health in persons with spinal cord injury (SCI).

**Methods:** We analyzed data from 12,588 participants of the International SCI Community Survey from 22 countries. To investigate individual-level inequalities, SES indicators (education, income, financial hardship, subjective status) were regressed on the SF-36 mental health index (MHI-5), stratified by countries. Country-level inequalities were analyzed with empirical Bayes estimates of random intercepts derived from linear mixed-models adjusting for individual-level SES.

**Results:** Financial hardship and subjective status consistently predicted individual-level mental health inequalities. Country-level SED was inconsistently related to mental health when adjusting for individual-level SES. It however appeared that higher SED was associated with better mental health within higher-resourced countries.

**Conclusion:** Reducing impoverishment and marginalization may present valuable strategies to reduce mental health inequalities in SCI populations. Investigations of country-level determinants of mental health in persons with SCI should consider influences beyond country-level SED, such as cultural factors.

## Introduction

With a prevalence of 10.7% in all world regions, mental disorders importantly contribute to the global burden of disease [1]. Mental disorders are even more frequent in individuals with physical impairments, exacerbating their individual burden of disease [2, 3]. As demonstrated in recent meta-analyses, the prevalence of depression and anxiety disorders in people with spinal cord injury (SCI) is, with 22.2% for depression [(95% confidence interval (CI) 18.7%–26.3%; total number of studies included (*k*) = 19, total number of participants (*n*) = 35,676)] and 27% for anxiety (95% CI 24%–30%; *k* = 18, *n* = 2,772), alarmingly high [4, 5]. Mental health status is largely shaped by social, economic, and environmental conditions as well as the availability of psychosocial resources. Persons with SCI might be particularly vulnerable to poor mental health due to an increased risk of experiencing stressors, such as discrimination, social exclusion, unemployment, and reduced availability of psychosocial resources [6–8]. It is therefore important to advance evidence on risk factors of poor mental health in persons with SCI to adequately tailor rehabilitative and public health interventions.

Several systematic reviews drawing on global evidence convincingly documented that low socioeconomic status (SES) presents a risk factor for poor mental health [3, 9–11]. It is assumed that socioeconomic disadvantage is linked to greater exposure to unfavorable psychosocial, behavioral and environmental conditions throughout the life course, ultimately negatively affecting people’s mental health [9]. Similar social inequalities in mental health were observed in SCI populations for depression [12–15], anxiety [12], and perceived mental health [16]. As various SES indicators reflect different sets of relevant resources for mental health, the distinction between distinct SES dimensions is pivotal to identify main drivers of poor mental health [3, 17]. The relative importance of different SES indicators with regard to mental health of persons with physical impairments is yet to be clarified as available evidence mainly relies on the traditional SES indicators education [13–16] and income [12–16], while studies on subjective dimensions of SES, such as perceived financial hardship [15, 16] and subjective status are rare.

Mental health may also depend on larger societal and structural conditions, such as a countries’ socioeconomic development (SED) as reflected by indicators such as gross domestic product or average life expectancy. These macro-level conditions possibly influence individual-level living conditions and social determinants which may ultimately affect mental health. For example, countries with greater income inequality and poorer developed welfare arrangements may provide less favorable environments for maintaining good mental health [18]. However, no cross-country comparative study on mental health in persons with physical impairments has been performed to date.

The present study investigates social inequalities in mental health of persons with SCI from 22 countries considering four distinct individual-level SES indicators (education, household income, financial hardship and subjective status) as well as country-level SED. More specifically, we aim to analyze 1) individual-level associations between different SES indicators and mental health within countries, and 2) between-country differences in average mental health that remain when individual-level SES is considered. In both cases, relationships with country-level SED are explored.

## Methods

### Design

This cross-sectional study uses data from the International SCI community survey (InSCI) performed in 22 countries between January 2017 and May 2019 covering all continents. In total, 12,588 community-dwelling persons with traumatic or non-traumatic SCI aged over 18 years participated in this survey. An injury to the spinal cord causes complete or partial loss of motor function and sensation below the lesion level, often severely affecting functioning and health [19]. Persons with neuro-degenerative disorders, congenital SCI etiologies, or Guillain Barré syndrome were excluded [20], as people with these health conditions usually follow different rehabilitation paths and disease progressions than those with acquired SCI and would thus present a non-comparable sub-population within the sample. National Study Centers were responsible for recruitment and data collection and sampling strategies varied according to local conditions, including random and convenience sampling. Countries offered paper-pencil or online questionnaires, telephone or personal interviews. Compliance with national laws and regulatory approvals by Institutional Review Boards or Ethical Committees was mandatory and the study conformed to the Helsinki Declaration. Informed consent was required from all participants according to national regulations. Details on methodological features, recruitment results, and participants characteristics are reported elsewhere [20, 21].

### Measures

*Mental health* was assessed with the SF-36 five-item Mental Health Index (MHI-5) [22] showing satisfactory reliability and validity as a screening instrument for general mental health in individuals with SCI [23]. The MHI-5 measures the frequency of experiencing five emotional states during the last month (0 = all of the time, 4 = none of the time). The raw sum score was transformed to a 0–100 score with higher scores indicating better mental health [22].

#### Individual-Level Socioeconomic Status

Education, income, financial hardship and subjective status were used as indicators for individual-level SES. Education was assessed as highest level of formal education obtained. For analysis this information was classified into three categories: no schooling/primary/lower secondary; upper/post-secondary; tertiary education. Given the high proportion of persons with no schooling or only primary education in China, Indonesia, Morocco and Thailand (28%–40% of samples), a classification differentiating no schooling/primary; lower/higher/post-secondary; and tertiary education was used for those countries. Net-equivalent household income in the countries’ currency was calculated by including information on household income, weighted by number of adults and children in the household according to criteria from the Organisation for Economic Co-Operation and Development (OECD) [24]. Given differences in the assessment of household income between countries (e.g., some before, some after taxes), we were unable to derive a measure that permitted cross-country comparison based on a common standard such as purchasing power parity. To nevertheless assess income inequalities within countries, income quartiles for each country were derived from income distributions within country samples. Financial hardship was evaluated with an item on the impact of people’s financial situation on their life during the past month (not applicable/no influence, made my life a little harder, made my life a lot harder) from the Nottwil Environmental Factors Inventory Short Form [25]. The MacArthur Scale of Subjective Social Status was used to assess subjective status on a 10-rung ladder [26], with higher values indicating higher subjective status.

#### Country-Level Socioeconomic Development

The Human Development Index (HDI) was used to operationalize country-level SED [27]. Developed by the United Nations, the HDI provides a summary measure of a country’s achievements along three dimensions: life expectancy at birth, access to knowledge represented as mean years of schooling for adults aged over 25 years and expected years of schooling for children of school-entering age, and standard of living represented as gross national income per capita in USD purchasing power parity. The HDI ranges from 0 to 1, with higher values indicating greater development. We used the HDI of 2017 (multiplied with 100 to receive legible effect sizes), the first year of the InSCI data collection [27].

#### Covariates

Gender, age, SCI severity (complete/incomplete paraplegia, complete/incomplete tetraplegia), etiology of SCI (traumatic, non-traumatic), years since injury, and mobility classes were considered as potential confounders. Mobility classes were derived from an item of the Spinal Cord Injury Independence Scale for self-report [28] with four categories: independent walking, walking with aids, manual wheelchair, power wheelchair/total assistance.

### Statistical Analysis

Analyses were conducted using STATA 16.0 for Windows (College Station, TX, United States). Associations of individual-level SES with mental health within countries were analyzed with linear regressions of mental health scores on SES predictors stratified by country. Two subsequent types of models were estimated: 1) unadjusted models in which mental health was regressed separately on each SES indicator; 2) adjusted models in which mental health was regressed simultaneously on all SES indicators and covariates. We report coefficients, 95% CI and *p*-values from global Wald-tests. Models were estimated both with individual-level SES predictors entered as factor variables (apart from subjective status) and as continuous variables. The latter served the investigation of linear trends. Coefficients representing linear trends for individual-level SES indicators within the different countries are plotted across countries sorted by HDI rank to identify obvious patterns.

To examine how far the cross-country differences in mental health were accounted for by between sample variation in average individual-level SES, sociodemographic, and SCI characteristics (covariates), we fitted a linear mixed-effects model with a random intercept for country and regressed mental health scores simultaneously on all individual-level SES indicators and covariates. We then preceded in two steps: First, covariate-adjusted marginal means and 95% CIs of the country-specific mental health scores as estimated from the mixed-effects model were plotted against HDI-rank of the countries. This shows the pattern of mental health by country HDI that we would expect to see if only differences in individual-level predictors (within and between countries) as estimated with the model’s fixed part mattered. Second, we used empirical Bayes estimation to derive best linear unbiased predictors (BLUPs) for random intercepts and their standard errors for each country [29]. These BLUPs represent the error in prediction due to unobserved between-country heterogeneity, i.e., residual variation in the data that is neither explained by the modeled individual factors nor accounted for by measurement error. These BLUPs can be interpreted as country-sample effects on the mental health of people with SCI, given covariates. BLUPs >0 indicate underestimation of the average mental health status with the fixed effects part of the model or over-performance of a country sample with regard to the mental health of people with SCI as compared to our expectation given cross-country variation in individual-level SES and covariates. BLUPs <0 indicate overestimation or underperformance of country samples with regard to expected country-level mental health. Estimated BLUPs of random intercepts with 95% CIs were plotted against country HDI. Restricted cubic-spline smoothening with 4 knots was used for exploration of patterns in the relationship of BLUPs and HDI [30]. R-squared and Root Mean-Squared Error of Approximation (RMSEA) are provided as indicators for goodness of fit of the restricted cubic-spline model to the data.

To assess potential bias due to missing values, analyses were repeated with complete and imputed data in sensitivity analysis. Missing values were imputed with multiple imputation (MI) by chained equations on 25 imputed datasets [31], assuming that data were missing at random. Results from both analyses were compared and no relevant differences between the two strategies were detected. Results shown are based on imputed data.

## Results

Table 1 provides details on basic sample characteristics. The majority of participants was male (73%), mean age was around 51 years (SD 15.3), incomplete paraplegia was the most (35%) and complete tetraplegia the least frequent SCI type (10%). In 80% of participants, SCI was caused by trauma. Average time since SCI was about 13 years (SD 11.9). Roughly two thirds of participants were wheelchair dependent. Around 18% indicated experiencing massive financial hardship and around 10% reported primary, 61% secondary, and 29% tertiary education as highest educational level. Average subjective status was 4.8 (SD 2.1). With the exception of income (which was represented in terms of country-sample quartiles), there was a trend for higher average individual-level SES in countries with greater HDI (see Supplementary Appendix S1 for country-specific SES distributions). For the 0–100 mental health scale overall average was 66.3 (SD 20.6). A detailed description of mental health scores in different countries can be found elsewhere (see Supplementary Table S2, [6]).

**TABLE 1:** Description of study variables in the 12,588 participants of the International Spinal Cord Injury community survey (22 countries, 2017–2019).

Variables [% missing values]	Total
**Categorical variables**	**N (%)**
Male gender [0.3]	9,165 (73.0)
SCI severity [4.3]	
Incomplete paraplegia	4,155 (34.5)
Complete paraplegia	3,381 (28.1)
Incomplete tetraplegia	3,284 (27.3)
Complete tetraplegia	1,225 (10.2)
Traumatic etiology [1.6]	9,990 (80.6)
Mobility classes [2.9]	
Walking without aids	2,036 (16.7)
Walking with aids	2,006 (16.4)
Manual wheelchair	5,392 (44.1)
Electric wheelchair/complete dependence	2,784 (22.8)
Highest level of education [2.3]	
No schooling or primary	1,183 (9.6)
Lower secondary	2,282 (18.6)
Upper or post-secondary	5,272 (42.9)
Tertiary	3,628 (29.3)
Net-equivalent household income [8.5]	
Lowest quartile	2,942 (25.6)
2nd lowest quartile	2,865 (24.9)
2nd highest quartile	2,903 (25.2)
Highest quartile	2,803 (24.4)
Financial hardship [4.0]	
Massive	2,156 (17.8)
Some	3,414 (28.2)
None	6,523 (53.9)
**Continuous variables**	**Mean (SD)**
Age in years [0.6]	51.3 (15.3)
Time since injury in years [2.7]	13.1 (11.9)
Subjective social status, 1–10 score [4.2]	4.8 (2.1)
Mental health MHI-5, 0–100 score [4.0]	66.3 (20.6)

Abbreviations: MHI-5, 5-item Mental Health Index; SCI, spinal cord injury; SD, standard deviation.

### Individual-Level Socioeconomic Status and Mental Health

**FIGURE 1:**
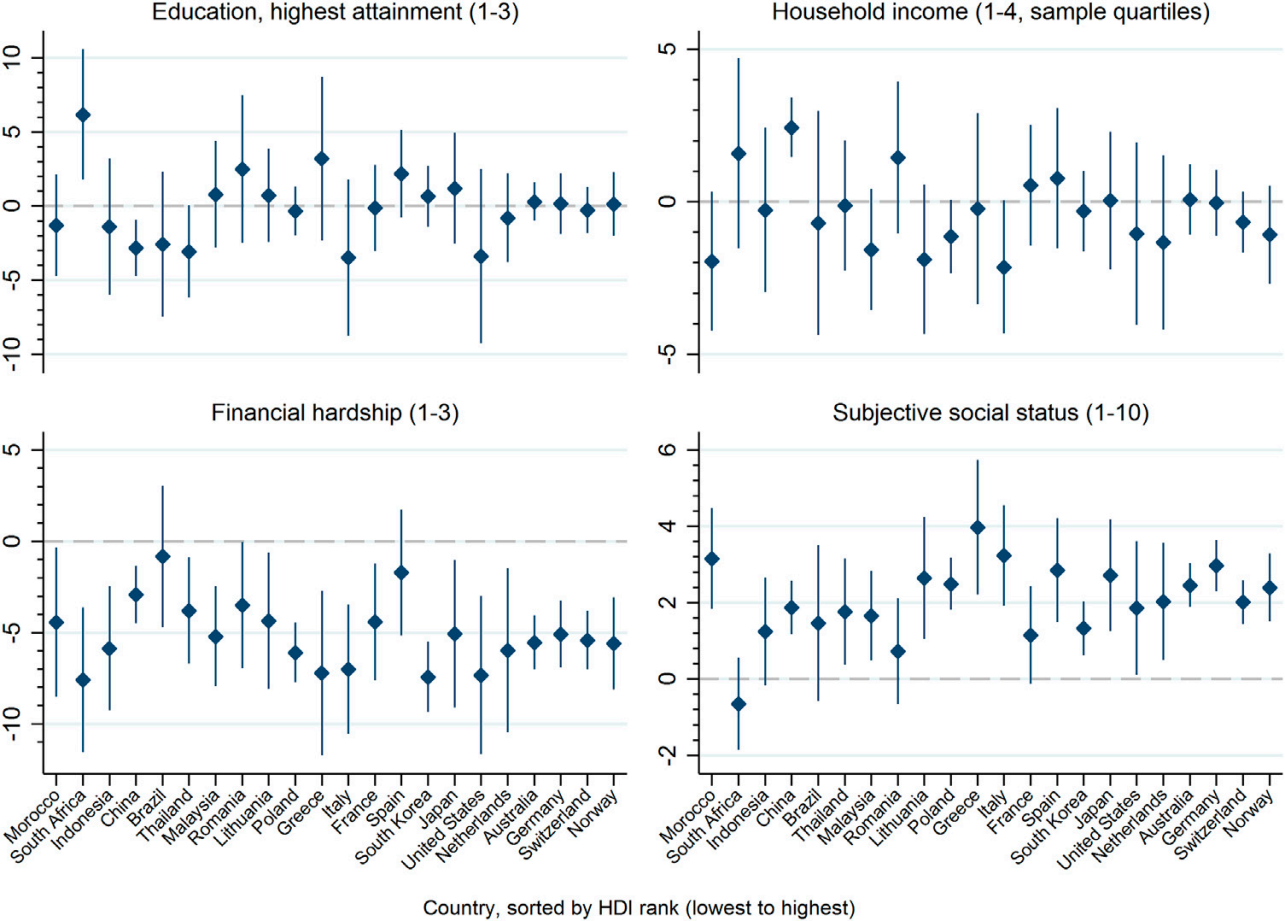
Adjusted coefficients and 95% confidence intervals from linear trends for associations of individual-level indicators of socioeconomic status (education, net-equivalent household income, financial hardship, subjective social status) with mental health (5-item Mental Health Index). Countries participating in the International Spinal Cord Injury community survey are sorted by their socioeconomic development operationalized by the Human Development Index (22 countries, 2017–2019).

Figure 1 shows adjusted coefficients of country-specific associations between individual-level SES and mental health (linear trends). Education and income were inconsistently associated with mental health and 95% CIs mostly included zero, indicating statistical non-significance. Exceptions are China (higher education related to worse mental health and higher income related to better mental health) and South Africa (higher education associated with better mental health). In contrast, mental health status decreased with the degree of perceived financial hardship in all countries, with statistically significant associations in 20 of 22 countries (adjusted models). Similarly, higher subjective status was linked to better mental health in all countries except South Africa, with statistically significant associations in 17 out of 22 countries (adjusted models). The results from models where education, income, and financial hardship were entered as factor variables largely corresponded to linear trends reported here (see Supplementary Appendixes S2, S3).

### Country-Level Socioeconomic Development and Mental Health

**FIGURE 2:**
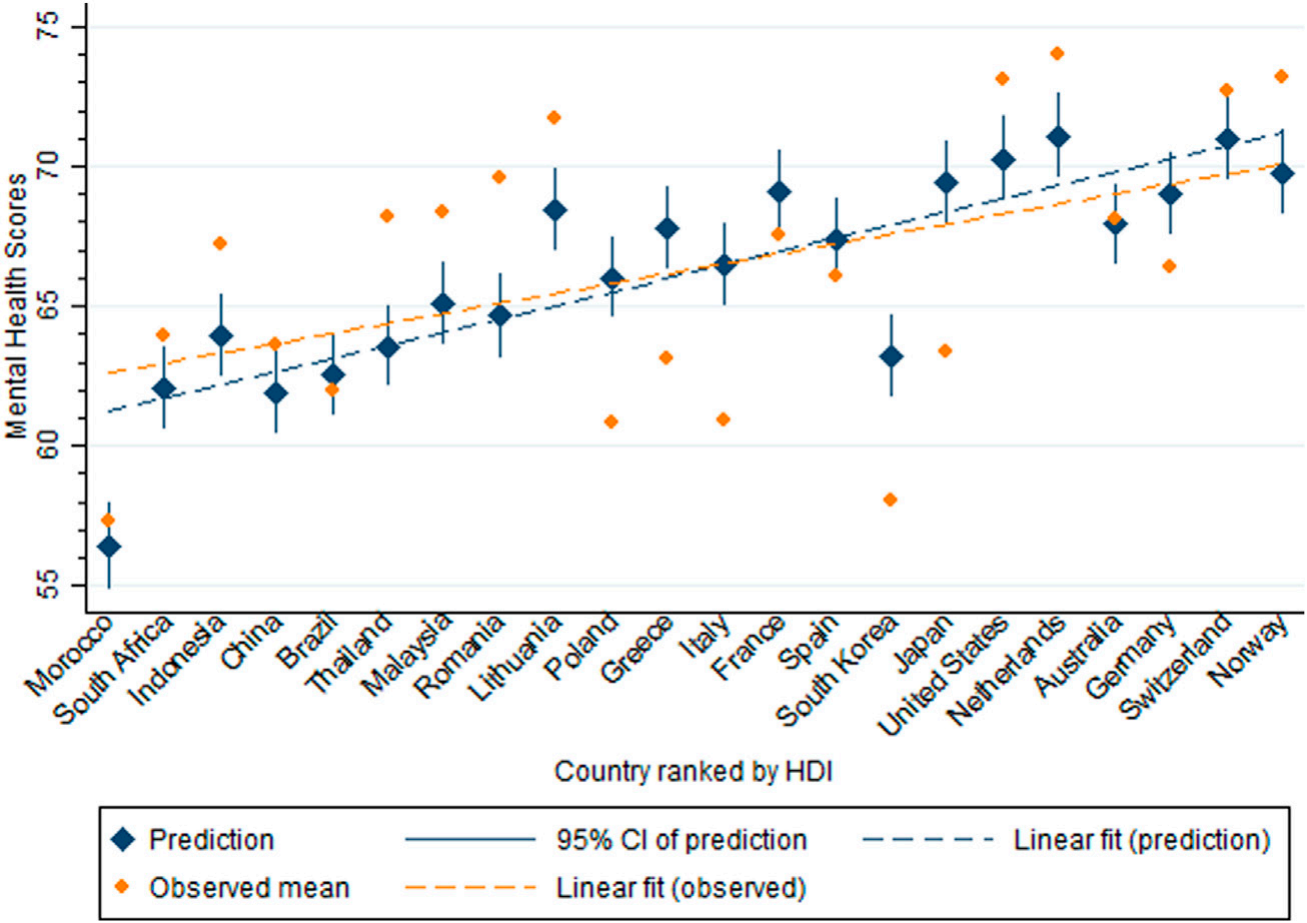
Observed and predicted mental health scores (5-item Mental Health Index) by countries participating in the International Spinal Cord Injury community survey, ordered according to their socioeconomic development operationalized by the Human Development Index (22 countries, 2017–2019).

Figure 2 shows observed and predicted marginal mental health scores plotted by countries HDI ranks. A clear trend towards better mental health in countries with higher HDI for mental health scores as predicted by the fixed effects part of the mixed model, stands in contrast to observed mental health scores in most countries. Against the predicted linear trend, Morocco and South Korea stand out for low and Lithuania for high predicted as well as observed mental health.

**FIGURE 3:**
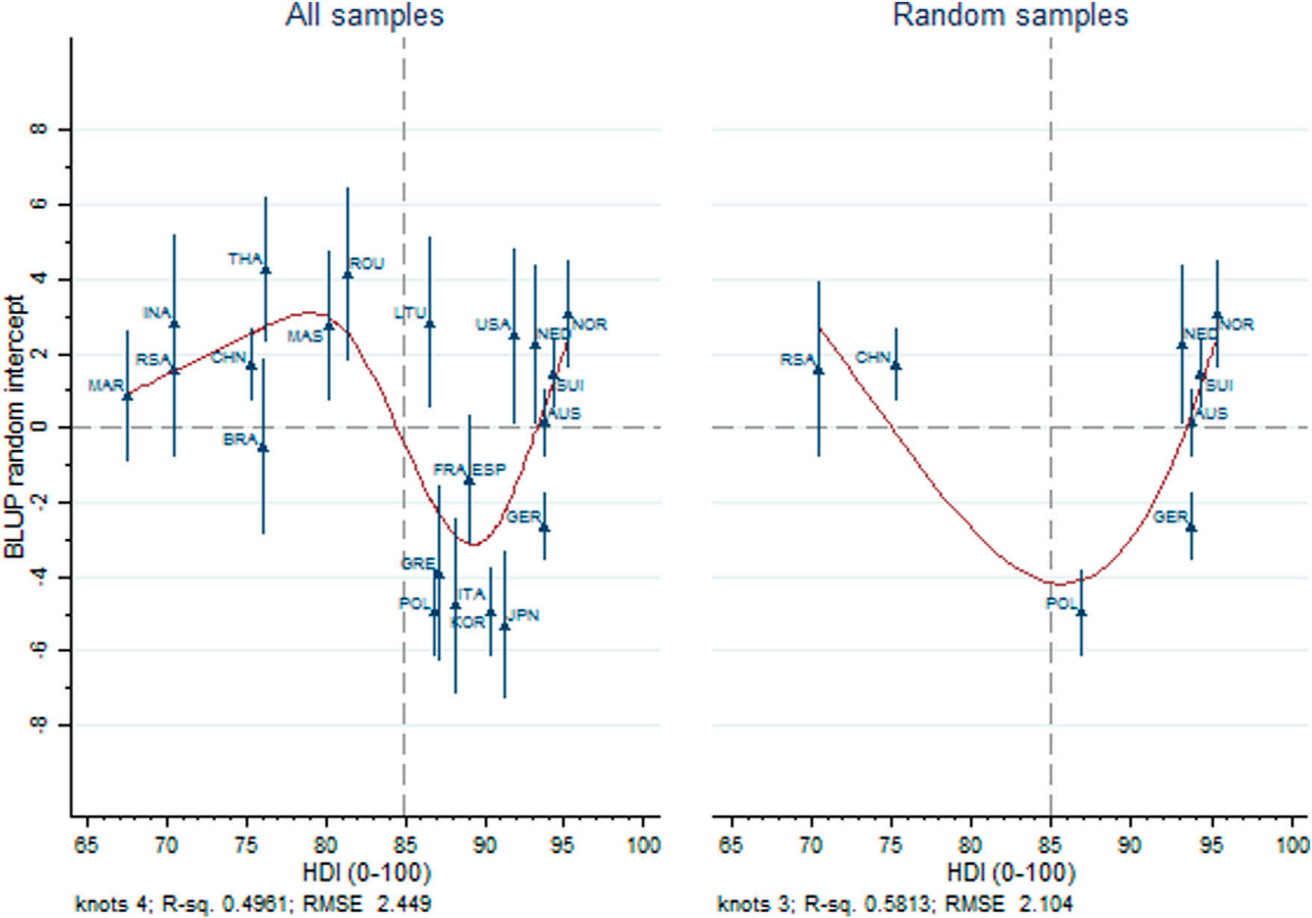
Estimated best linear unbiased predictions for random intercepts and their standard errors from mixed effects models regressing mental health on individual-level socioeconomic status and covariates, from all samples and only for countries participating in the International Spinal Cord Injury community survey using random sampling. The dashed vertical line indicates overall average of the Human Development Index of included country samples (22 countries, 2017–2019).

Figure 3 displays BLUPs for country-specific random intercepts, representing between-country variation unaccounted for individual-level SES and covariates. All countries with an HDI below overall average (84.9, vertical dashed line) apart from Brazil show better mental health than expected based on individual-level SES and covariates. In contrast, 8 out of 14 countries with above-average HDI perform worse than expected. The restricted cubic spline smoothening model explained 48.7% of the variance of random intercepts for all samples and 58.9% when only considering countries with random samples. We observed different patterns for below- vs. above-average HDI countries in both cases. When considering all countries, below-average HDI countries show a trend of increasingly positive residual country-level effects on mental health that corresponds to increasing HDI. The curve then sharply declines between Romania and Poland and increases sharply again afterwards in approximately linear fashion. This increase from countries with worse to those with better mental health than the expectation derived from estimated effects of individual-level predictors again corresponds to increased HDI. The one exception not fitting this pattern is Lithuania. The latter trend is equally obvious in the graph displaying countries with random samples only, while no such relation is observed for the two remaining countries with below-average HDI.

## Discussion

This study provides initial evidence for social inequalities of mental health within a population of people with physical impairments due to SCI. We observed that subjective indicators of SES (financial hardship and subjective status) were consistently related to poorer mental health whereas effects of the traditional SES indicators education and income on mental health were more volatile across countries and mostly statistically insignificant. These findings highlight the importance of including subjective SES indicators in research that aims to identify key drivers of poor mental health in people with SCI. While average individual-level SES of people with SCI was generally lower in countries with lower country-level SED as reflected by the HDI, cross-country differences in mental health were neither sufficiently explained by this variation in individual-level SES nor by differences in sample composition regarding sociodemographic and SCI-related characteristics. Residual between-country variation in average mental health also did not clearly follow differences in country-level SED. A group of countries with lower SED (below-average HDI) was clearly set apart from a group with higher SED. The observed mental health of people with SCI from countries with below-average HDI was generally better than predictions based on individual-level SES and covariates would suggest. Nonetheless, in both, countries with below- as well as above-average HDI, increasing HDI appeared to be related to better mental health. This trend was, however, only confirmed for countries with above-average HDI when only countries using random sampling frames for recruitment were considered.

In contrast to other studies in SCI populations [12–16], our country-specific results showed that education and income had limited predictive value for mental health in comparison to more subjective SES indicators. This is in line with previous findings from general population surveys showing that subjective status was more strongly linked to health outcomes than traditional SES indicators [32, 33]. Low subjective status may reflect feelings of marginalization and social exclusion better than objective SES indicators and thus more readily trigger negative emotions that ultimately obstruct mental health. Perceived financial hardship may more adequately capture conditions of poverty where ends cannot be met than nominal income. Perceived financial hardship may affect mental health through daily hassles and psychosocial distress [34, 35]. This may be of particular relevance in a population with increased healthcare needs and costs due to physical impairment. Perceived financial hardship might also be particularly harmful for the mental health of persons with physical impairments as it may lead to restricted social participation and social exclusion [16, 36]. Also, poorer access to health care services in general, and psychological support services in particular, might negatively affect mental health in persons experiencing financial hardship [9].

Our study further demonstrated a statistical association between county-level SED and mental health at the population level. However, this association was less straightforward than it might be expected. Cross-country differences in mental health were neither explained by between-country variation in individual-level SES, nor was there an overall linear trend for the relation of country HDI with residual between-country differences in mental health scores. In contrast, we observed separate trends for countries with below- and above-average HDI. Only for countries with above-average HDI (higher SED countries), higher HDI was robustly associated with better mental health. For these countries, increased availability of mental health services and access to psychological support might drive inequalities in mental health at the country-level [9]. In turn, countries with below-average HDI showed a better country-level performance in mental health than a majority of higher HDI countries given variation in individual-level SES and covariates. Within all below-average HDI countries, increasing HDI then appeared to be related to better mental health as well. However, the latter finding was not robust when only countries with random samples were considered, possibly due to the low number of countries with lower SED that employed such sampling frame. These patterns in cross-country differences in mental health and their association with SED may reflect different distributions of self-reported mental health in different groups of countries, i.e. those with below- vs. above-average HDI. We subsequently discuss four potential explanations for the observed associations. First, problems in mental health might be underreported in countries where mental disorders have historically been a taboo and/or associated with stigmatization [37]. Second, recall bias and a stronger focus on current affect state is conceivable as receiving attention through the survey may have improved the affect state of people from countries where such surveys are uncommon. Third, the HDI does not consider dimensions relevant to people’s mental health that are in the favor of lower resource countries, such as stronger family cohesion or collective value-orientation. Fourth, important (unobserved) individual-level variables that show strong variation between countries and have a strong effect on mental health have been omitted from the model, e.g. perceived family support or availability of informal caregivers.

### Implications

Given that poor mental health, financial hardship and low subjective status interact bi-directionally, interventions may not exclusively target mental health outcomes through psychological interventions, but also consider acting on the reduction of financial hardship or poverty and the personal perception of one’s standing in society [38]. Interventions may address barriers limiting individual and collective opportunities for economic integration and participation [39], e.g., labor market access and increased social participation for persons with physical impairments. Besides attempting to reduce poverty and marginalization in persons with SCI, the removal of economic and socio-cultural barriers and systemic discrimination as well as strengthening specific psychosocial resources in lower SES groups may present promising strategies to support mental health equality in persons with SCI. On the country-level, it should be further scrutinized what factors beyond SED as measured with common indices like the HDI possibly contribute to mental health.

### Limitations

The representativeness of the study samples to the total population of individuals with SCI in the participating countries and thus the generalizability of the results is limited as 14 of 22 countries relied on convenience sampling, while only eight countries applied random sampling strategies. It is also worthwhile mentioning that the results for specific countries cannot be generalized to respective continents, as the within-continent differences in SED is in some cases relevant (e.g., southern Asian countries vs. other Asian countries). Moreover, the operationalization of income as quartiles of the within country-sample distribution may is suboptimal as those quartiles only reflect the income situation in relation to other people with SCI in the same country-sample and not their income situation in relation to the total population. Moreover, cross-country differences in purchasing power cannot be captured in this way. The lack of information on mental health or SES for non-responders prevents us from assessing whether non-response was patterned according to those characteristics. We further cannot evaluate whether self-report on mental health or SES led to biased responses, as for example information on income and mental health are prone to social desirability bias, particularly if poverty and/or mental diseases are subject to stigmatization. Also, it remains unclear whether the different data collection methods introduced any bias in responses. Finally, reverse causation of associations cannot be excluded, especially for the indicator subjective status, as persons with poor mental health might be prone to evaluate their social status as low.

### Conclusion

This study provides evidence for social inequalities in mental health in persons with SCI across different regions of the world, highlighting the importance of perceived financial hardship and low subjective status as predictors of decreased mental health. Conversely, the traditional SES indicators education and income poorly explained differences in mental health and showed inconsistent effects across countries. There was no clear overall association of country-level SED with average mental health of SCI populations. It appears, however, that higher levels of SED are associated with better mental health within the group of higher-resourced countries. Efforts to reduce financial hardship and to increase the subjective status of people with SCI present promising strategies to reduce mental health inequalities in this population within and across countries.

## References

[1] James S Abate D Abate K Abay S Abbafati C Abbasi N Global, Regional, and National Incidence, Prevalence, and Years Lived with Disability for 354 Diseases and Injuries for 195 Countries and Territories, 1990–2017: a Systematic Analysis for the Global Burden of Disease Study 2017. Lancet (2017) 392(10159):1789–858. 10.1016/S0140-6736(18)32279-7 PMC6227754 30496104

[2] Moussavi S Chatterji S Verdes E Tandon A Patel V Ustun B. Depression, Chronic Diseases, and Decrements in Health: Results from the World Health Surveys. Lancet (2007) 370(9590):851–8. 10.1016/S0140-6736(07)61415-9 17826170

[3] Pinto-Meza A Moneta MV Alonso J Angermeyer MC Bruffaerts R Caldas de Almeida JM Social Inequalities in Mental Health: Results from the EU Contribution to the World Mental Health Surveys Initiative. Soc Psychiatry Psychiatr Epidemiol (2013) 48(2):173–81. 10.1007/s00127-012-0536-3 23011445

[4] Williams R Murray A. Prevalence of Depression after Spinal Cord Injury: a Meta-Analysis. Arch Phys Med Rehabil (2015) 96(1):133–40. 10.1016/j.apmr.2014.08.016 25220943

[5] Le J Dorstyn D. Anxiety Prevalence Following Spinal Cord Injury: a Meta-Analysis. Spinal Cord (2016) 54(8):570–8. 10.1038/sc.2016.15 26951738

[6] Fekete C Tough H Arora M Hasnan N Joseph C Popa D Are Social Relationships an Underestimated Resource for Mental Health in Persons Experiencing Disability? Observational Evidence from 22 Countries. Int J Public Health (2021) 66:619823. 10.3389/ijph.2021.619823 34744581 PMC8565297

[7] Reinhardt JD Middleton J Bokel A Kovindha A Kyriaides A Hajjioui A Environmental Barriers Experienced by People with Spinal Cord Injury across 22 Countries: Results from a Cross-Sectional Survey. Arch Phys Med Rehabil (2020) 101(12):2144–56. 10.1016/j.apmr.2020.04.027 32502565

[8] Carrard V Kunz S Peter C. Mental Health, Quality of Life, Self-Efficacy, and Social Support of Individuals Living with Spinal Cord Injury in Switzerland Compared to that of the General Population. Spinal Cord (2021) 59(4):398–409. 10.1038/s41393-020-00582-5 33235298

[9] World Health Organization. Social Determinants of Mental Health. Geneva, Switzerland: World Health Organization (2014).

[10] Lorant V Deliege D Eaton W Robert A Philippot P Ansseau M. Socioeconomic Inequalities in Depression: a Meta-Analysis. Am J Epidemiol (2003) 157(2):98–112. 10.1093/aje/kwf182 12522017

[11] Silva M Loureiro A Cardoso G. Social Determinants of Mental Health: a Review of the Evidence. Eur J Psychiat (2016) 30:259–92. Available at: https://scielo.isciii.es/pdf/ejpen/v30n4/original03.pdf (Accessed November 19, 2022).

[12] Lim SW Shiue YL Ho CH Yu SC Kao PH Wang JJ Anxiety and Depression in Patients with Traumatic Spinal Cord Injury: a Nationwide Population-Based Cohort Study. PloS one (2017) 12(1):e0169623. 10.1371/journal.pone.0169623 28081205 PMC5231351

[13] Krause JS Kemp B Coker J. Depression after Spinal Cord Injury: Relation to Gender, Ethnicity, Aging, and Socioeconomic Indicators. Arch Phys Med Rehabil (2000) 81(8):1099–109. 10.1053/apmr.2000.7167 10943762

[14] Khazaeipour Z Taheri-Otaghsara SM Naghdi M. Depression Following Spinal Cord Injury: its Relationship to Demographic and Socioeconomic Indicators. Top Spinal Cord Inj Rehabil (2015) 21(2):149–55. 10.1310/sci2102-149 26364284 PMC4568096

[15] Zurcher C Tough H Fekete C. Mental Health in Individuals with Spinal Cord Injury: The Role of Socioeconomic Conditions and Social Relationships. PloS one (2019) 14(2):e0206069. 10.1371/journal.pone.0206069 30785880 PMC6382129

[16] Fekete C Siegrist J Reinhardt JD Brinkhof MW. Is Financial Hardship Associated with Reduced Health in Disability? the Case of Spinal Cord Injury in Switzerland. PloS one (2014) 9(2):e90130. 10.1371/journal.pone.0090130 24587239 PMC3938582

[17] Lund C Breen A Flisher AJ Kakuma R Corrigall J Joska JA Poverty and Common Mental Disorders in Low and Middle Income Countries: A Systematic Review. Soc Sci Med (2010) 71(3):517–28. 10.1016/j.socscimed.2010.04.027 20621748 PMC4991761

[18] Ploubidis GB Grundy E. Later-life Mental Health in Europe: a Country-Level Comparison. J Gerontol B Psychol Sci Soc Sci (2009) 64(5):666–76. 10.1093/geronb/gbp026 19414867

[19] Bickenbach J Officer A Shakespeare T von Groote P. International Perspectives on Spinal Cord Injury. Geneva, Switzerland: World Health Organization (2013).

[20] Gross-Hemmi MH Post MW Ehrmann C Fekete C Hasnan N Middleton JW Study Protocol of the International Spinal Cord Injury (InSCI) Community Survey. Am J Phys Med Rehabil (2017) 96:S23–S34. 10.1097/PHM.0000000000000647 28059876

[21] Fekete C Brach M Ehrmann C Post MW Stucki G. Cohort Profile of the International Spinal Cord Injury Community Survey Implemented in 22 Countries. Arch Phys Med Rehabil (2020) 101(12):2103–11. 10.1016/j.apmr.2020.01.022 32533933

[22] Ware J Snow K Kostinski M Gandek B. SF-36 Health Survey - Manual and Interpretation Guide. Boston, USA: The Health Institute, New England Medical Center (1993).

[23] van Leeuwen CM van der Woude LH Post MW. Validity of the Mental Health Subscale of the SF-36 in Persons with Spinal Cord Injury. Spinal Cord (2012) 50(9):707–10. 10.1038/sc.2012.33 22487956

[24] Hagenaars A de Vos K Zaidi MA. Poverty Statistics in the Late 1980s: Research Based on Micro-data. Luxembourg: Statistical Office of the European Communities (1994).

[25] Ballert CS Post MW Brinkhof MW Reinhardt JD. Psychometric Properties of the Nottwil Environmental Factors Inventory Short Form. Arch Phys Med Rehabil (2015) 96(2):233–40. 10.1016/j.apmr.2014.09.004 25264112

[26] Adler N Stewart J. The MacArthur Scale of Subjective Social Status (2007). Available from: http://www.macses.ucsf.edu/research/psychosocial/subjective.php (Accessed December 10 2021).

[27] United Nations Development Program. Human Development Index (2019). Available from: http://hdr.undp.org/en/content/human-development-index-hdi (Accessed December 10 2021).

[28] Fekete C Eriks-Hoogland I Baumberger M Catz A Itzkovich M Luthi H Development and Validation of a Self-Report Version of the Spinal Cord Independence Measure (SCIM III). Spinal Cord (2013) 51(1):40–7. 10.1038/sc.2012.87 22890418

[29] Rabe-Hesketh S Skrondal A. Multilevel and Longitudinal Modeling Using Stata. 4th ed. College Station, TX: Stata Press (2021).

[30] Eilers P Marx B. Splines, Knots, and Penalties. Wires Comp Stat (2010) 2(6):637–53. 10.1002/wics.125

[31] White IR Royston P Wood AM. Multiple Imputation Using Chained Equations: Issues and Guidance for Practice. Stat Med (2011) 30:377–99. 10.1002/sim.4067 21225900

[32] Singh-Manoux A Marmot MG Adler NE. Does Subjective Social Status Predict Health and Change in Health Status Better Than Objective Status? Psychosom Med (2005) 67(6):855–61. 10.1097/01.psy.0000188434.52941.a0 16314589

[33] Adler NE Epel ES Castellazzo G Ickovics JR. Relationship of Subjective and Objective Social Status with Psychological and Physiological Functioning: Preliminary Data in Healthy white Women. Health Psychol (2000) 19(6):586–92. 10.1037//0278-6133.19.6.586 11129362

[34] Bradshaw M Ellison CG. Financial Hardship and Psychological Distress: Exploring the Buffering Effects of Religion. Soc Sci Med (2010) 71(1):196–204. 10.1016/j.socscimed.2010.03.015 20556889 PMC3770858

[35] Frasquilho D de Matos MG Marques A Gaspar T Caldas-de-Almeida JM. Distress and Unemployment: the Related Economic and Noneconomic Factors in a Sample of Unemployed Adults. Int J Public Health (2016) 61(7):821–8. 10.1007/s00038-016-0806-z 26971795

[36] Gross-Hemmi MH Post MWM Bienert S Chamberlain JD Hug K Jordan X Participation in People Living with Spinal Cord Injury in Switzerland: Degree and Associated Factors. Arch Phys Med Rehabil (2019) 100(10):1894–906. 10.1016/j.apmr.2019.03.018 31026462

[37] Kitanaka J Ecks S Yi-Jui Wu H. The Social in Psychiatries: Depression in Myanmar, China, and Japan. Lancet (2021) 398(10304):948–9. 10.1016/S0140-6736(21)00999-5 34058132

[38] Lund C De Silva M Plagerson S Cooper S Chisholm D Das J Poverty and Mental Disorders: Breaking the Cycle in Low-Income and Middle-Income Countries. Lancet (2011) 378(9801):1502–14. 10.1016/S0140-6736(11)60754-X 22008425

[39] Burns JK. Poverty, Inequality and a Political Economy of Mental Health. Epidemiol Psychiatr Sci (2015) 24(2):107–13. 10.1017/S2045796015000086 25746820 PMC6998107

