# Supplementary material for: Socioeconomic Status, the Countries’ Socioeconomic Development and Mental Health: Observational Evidence for Persons with Spinal Cord Injury from 22 Countries
Source: Int J Public Health. 2022 Nov 30;67:1604673. doi: 10.3389/ijph.2022.1604673 (PMC9747630; doi:10.3389/ijph.2022.1604673)
Supplement: Supplementary file 1 [file DataSheet3.DOCX]

**Supplementary Table 3.** Adjusted association of socioeconomic status and mental health: β-coefficients and 95% confidence intervals from linear regressions, stratified by the 22 countries participating in the International Spinal Cord Injury community survey (22 countries, 2017-2019).

|  | **Mental health**  (MHI-5 score 0-100, higher scores=better mental health) | | | | | | | | | | | | | | | | | | | | | | | | | | | | | | | | | | | | | | | | | | | |
| --- | --- | --- | --- | --- | --- | --- | --- | --- | --- | --- | --- | --- | --- | --- | --- | --- | --- | --- | --- | --- | --- | --- | --- | --- | --- | --- | --- | --- | --- | --- | --- | --- | --- | --- | --- | --- | --- | --- | --- | --- | --- | --- | --- | --- |
|  | Australia (n=1579) | | Brazil  (n=201) | | China  (n=1354) | | France (n=412) | | Germany (n=1617) | | Greece (n=200) | | Indonesia (n=201) | | Italy  (n=206) | | Japan (n=302) | | Lithuania (n=218) | | Malaysia  (n=297) | | Morocco (n=385) | | Netherlands (n=260) | | Norway (n=609) | | Poland (n=971) | | Romania  (n=216) | | South Africa (n=200) | | South Korea (n=890) | | Spain  (n=417) | | Switzerland (n=1530) | | Thailand (n=320) | | United States (n=203) | |
|  | Coeff  95% CI | | Coeff  95% CI | | Coeff  95% CI | | Coeff  95% CI | | Coeff  95% CI | | Coeff  95% CI | | Coeff  95% CI | | Coeff  95% CI | | Coeff  95% CI | | Coeff  95% CI | | Coeff  95% CI | | Coeff  95% CI | | Coeff  95% CI | | Coeff  95% CI | | Coeff  95% CI | | Coeff  95% CI | | Coeff  95% CI | | Coeff  95% CI | | Coeff  95% CI | | Coeff  95% CI | | Coeff 95% CI | | Coeff 95% CI | |
| **Education** |  | |  | |  | |  | |  | |  | |  | |  | |  | |  | |  | |  | |  | |  | |  | |  | |  | |  | |  | |  | |  | |  | |
| No/primary | Ref | | Ref | | Ref | | Ref | | Ref | | Ref | | Ref | | Ref | | Ref | | Ref | | Ref | | Ref | | Ref | | Ref | | Ref | | Ref | | Ref | | Ref | | Ref | | Ref | | Ref | | *Ref* | |
| Secondary | 1.20  -1.29-3.69 | | -4.05  -12.01-3.91 | | -3.82  -6.03- -1.62 | | 1.31  -3.84-6.47 | | 2.83  -1.17-6.83 | | 6.01  -2.52-14.54 | | -0.62  -6.46-5.22 | | -6.50  -14.55-1.56 | | 1.14  -5.14-7.43 | | 1.28  -6.37-8.92 | | -0.94  -7.71-5.84 | | 1.50  -3.71-6.71 | | -0.52  -6.75-5.71 | | 2.77  -1.40-6.95 | | -0.54  -3.18-2.10 | | 0.34  -18.29-18.96 | | 7.47  1.12-13.82 | | 0.64  -3.38-4.65 | | 2.46  -3.61-8.53 | | 0.34  -2.77-3.45 | | -3.07  -8.42-2.27 | | 14.20  -15.78-44.18 | |
| Tertiary | 0.52  -2.07-3.11 | | -6.53  -17.01-3.95 | | -3.64  -7.96-0.068 | | -0.42  -6.32-5.49 | | 1.68  -2.79-6.16 | | 5.85  -5.12-16.81 | | -2.12  -12.17-7.94 | | -7.10  -17.66-3.46 | | 2.67  -4.93-10.27 | | -0.08  -6.30-6.14 | | 1.25  -6.38-8.89 | | -3.22  -10.28-3.85 | | -1.84  -7.93-4.25 | | 0.89  -3.59-5.37 | | -0.15  -3.53-3.22 | | 3.25  -15.06-21.57 | | 10.30  1.27-19.33 | | 1.37  -2.94-5.68 | | 4.58  -1.38-10.54 | | -0.26  -3.72-3.20 | | -6.41  -12.70- -0.12 | | 10.46  -19.23-40.15 | |
| Linear trends | 0.30  -1.00-1.59 | | -2.55  -7.43-2.32 | | -2.81  -4.71- -0.92 | | -0.11  -3.02-2.80 | | 0.18  -1.87-2.22 | | 3.21  -2.31-8.74 | | -1.38  -5.98-3.21 | | -3.48  -8.74-1.78 | | 1.21  -2.54-4.96 | | 0.72  -2.42-3.87 | | 0.80  -2.78-4.39 | | -1.30  -4.71-2.12 | | -0.78  -3.78-2.21 | | 0.13  -2.00-2.27 | | -0.33  -1.98-1.32 | | 2.50  -2.48-7.47 | | 6.18  1.78-10.59 | | 0.67  -1.39-2.73 | | 2.18  -0.76-5.13 | | -0.27  -1.83-1.29 | | -3.07  -6.17-0.03 | | -3.39  -9.26-2.49 | |
| *p-value* | *0.631* | | *0.432* | | *0.003* | | *0.720* | | *0.283* | | *0.373* | | *0.917* | | *0.274* | | *0.777* | | *0.930* | | *0.673* | | *0.355* | | *0.828* | | *0.301* | | *0.917* | | *0.572* | | *0.033* | | *0.804* | | *0.318* | | *0.838* | | *0.135* | | *0.358* | |
| **Household income** |  | |  | |  | |  | |  | |  | |  | |  | |  | |  | |  | |  | |  | |  | |  | |  | |  | |  | |  | |  | |  | |  | |
| Lowest quartile | Ref | | Ref | | Ref | | Ref | | Ref | | Ref | | Ref | | Ref | | Ref | | Ref | | Ref | | Ref | | Ref | | Ref | | Ref | | Ref | | Ref | | Ref | | Ref | | Ref | | Ref | | Ref | |
| 2^nd^ lowest quartile | 1.89  -1.06-4.83 | | 1.57  -7.17-10.31 | | 1.96  -0.72-4.65 | | -1.31  -6.66-4.04 | | 1.00  -1.98-3.98 | | -11.56  -20.70- -2.42 | | 6.11  -0.84-13.06 | | 0.58  -6.28-7.44 | | -4.19  -10.55-2.18 | | 3.95  -3.41-11.31 | | -1.76  -7.46-3.94 | | -1.50  -7.77-4.77 | | -0.51  -8.09-7.08 | | -2.43  -6.45-1.59 | | -2.83  -6.11-0.44 | | 1.54  -5.50-.58 | | -3.46  -11.81-4.90 | | 1.69  -2.45-5.83 | | -5.37  -11.86-1.11 | | -1.75  -4.67-1.18 | | 0.27  -5.79-6.33 | | -2.00  -9.30-5.30 | |
| 2^nd^ highest quartile | 0.26  -2.75-3.46 | | -0.58  -10.62-9.47 | | 7.62  4.82-10.43 | | -2.90  -8.89-3.08 | | -0.47  -3.61-2.68 | | 0.22  -9.00-9.44 | | 4.17  -3.26-11.60 | | -2.91  -9.61-3.78 | | -2.17  -9.24-4.90 | | 4.94  -2.59-12.47 | | -4.03  -9.88-1.82 | | -0.39  -6.92-6.14 | | -4.43  -12.57-3.70 | | -3.82  -8.39-0.74 | | -0.87  -4.20-2.46 | | 5.94  -0.96-12.85 | | 2.02  -6.10-10.13 | | -0.43  -4.31-3.44 | | 1.76  -4.92-8.44 | | -1.27  -4.24-1.69 | | -1.00  -7.59-5.58 | | -5.59  -12.88-1.70 | |
| Highest quartile | 0.76  -2.88-4.40 | | -0.65  -12.69-11.40 | | 6.04  2.96-9.11 | | 2.00  -4.20-8.20 | | 0.54  -2.78-3.87 | | -6.00  -15.79-3.79 | | -0.36  -8.88-8.17 | | -6.18  -13.03-0.67 | | -0.44  -7.53-6.65 | | -5.27  -12.78-2.24 | | -4.04  -10.26-2.19 | | -7.40  -14.50- -0.29 | | -3.82  -12.55-4.90 | | -3.02  -7.99-1.95 | | -4.57  -8.37- -0.77 | | 2.54  -5.55-10.63 | | 5.58  -4.92-16.09 | | -0.29  -4.37-3.80 | | -0.78  -8.03-6.47 | | -2.48  -5.68-0.71 | | 0.02  -6.68-6.73 | | -2.12  -12.01-7.78 | |
| Linear trends | 0.07  -1.07-1.22 | | -0.69  -4.36-2.98 | | 2.44  1.46-3.41 | | 0.54  -1.44-2.52 | | -0.04  -1.11-1.04 | | -0.22  -3.35-2.90 | | -0.27  -2.97-2.44 | | -2.14  -4.32-0.04 | | 0.04  -2.21-2.30 | | -1.89  -4.34-0.56 | | -1.56  -3.55-0.42 | | -1.95  -4.22-0.33 | | -1.33  -4.18-1.53 | | -1.08  -2.69-0.53 | | -1.13  -2.33-0.07 | | 1.46  -1.04-3.95 | | 1.59  -1.53-4.71 | | -0.31  -1.62-1.01 | | 0.77  -1.52-3.07 | | -0.67  -1.66-0.33 | | -0.12  -2.25-2.01 | | -1.04  -4.03-1.95 | |
| *p-value* | *0.607* | | *0.960* | | *<0.001* | | *0.371* | | *0.771* | | *0.030* | | *0.187* | | *0.218* | | *0.555* | | *0.023* | | *0.493* | | *0.136* | | *0.636* | | *0.419* | | *0.060* | | *0.380* | | *0.406* | | *0.780* | | *0.151* | | *0.437* | | *0.978* | | *0.493* | |
| **Financial hardship** |  | |  | |  | |  | |  | |  | |  | |  | |  | |  | |  | |  | |  | |  | |  | |  | |  | |  | |  | |  | |  | |  | |
| Massive | Ref | | Ref | | Ref | | Ref | | Ref | | Ref | | Ref | | Ref | | Ref | | Ref | | Ref | | Ref | | Ref | | Ref | | Ref | | Ref | | Ref | | Ref | | Ref | | Ref | | Ref | | Ref | |
| Some | 7.90  4.58-11.23 | | 3.55  -5.03-12.14 | | 5.16  2.66-7.66 | | 5.18  -1.88-12.24 | | 5.73  1.65-9.81 | | 13.78  3.89-23.67 | | 6.23  0.21-12.26 | | 5.24  -2.18-12.67 | | 9.81  0.04-19.57 | | 13.93  6.43-21.44 | | 8.80  3.27-14.33 | | 3.79  -1.65-9.23 | | 12.42  0.51-24.33 | | 6.19  0.10-12.28 | | 7.52  4.34-10.70 | | 3.95  -2.67-10.57 | | 1.99  -4.37-8.34 | | 10.64  0.62-2.03 | | -3.24  -11.00-4.51 | | 4.50  0.62-8.39 | | 1.90  -3.70-7.51 | | 3.04  -7.15-13.22 | |
| None | 11.88  8.76-15.00 | | 2.03  -5.96-10.01 | | 6.64  3.49-9.79 | | 9.57  2.64-16.51 | | 10.31  6.38-14.24 | | 17.63  8.20-27.06 | | 12.66  5.83-19.49 | | 13.46  5.90-21.02 | | 12.36  3.13-21.60 | | 14.60  6.52-22.67 | | 10.84  5.16-16.51 | | 11.37  1.57-21.17 | | 15.31  4.58-26.04 | | 11.36  5.67-12.28 | | 12.51  9.21-15.80 | | 6.99  0.06-13.92 | | 16.04  7.67-24.41 | | 14.77  10.90-18.63 | | 2.28  -5.12-9.67 | | 10.51  6.96-14.05 | | 7.27  1.37-13.18 | | 12.54  2.74-22.35 | |
| Linear trends | -5.53  -7.01- -4.05 | | -0.82  -4.69-3.05 | | -2.91  -4.48- 1.34 | | -4.41  -7.61- -1.21 | | -5.07  -6.91- -3.24 | | -7.21  -11.72- -2.70 | | -5.86  -9.25- -2.47 | | -7.00  -10.55- -3.46 | | -5.06  -9.10- -1.03 | | -4.35  -8.09- -0.60 | | -5.20  -7.94- -2.45 | | -4.44  -8.53- -0.35 | | -5.97  -10.47- -1.47 | | -5.59  -8.11- -3.07 | | -6.09  -7.71- -4.46 | | -3.48  -6.94- -0.02 | | -7.42  -11.54- 3.63 | | -7.42  -9.35- -5.49 | | -1.71  -5.15-1.74 | | -5.40  -7.00- -3.80 | | -3.79  -6.70- -0.87 | | -7.33  -11.66- -2.99 | |
| *p-value* | *<0.001* | | *0.715* | | *<0.001* | | *<0.001* | | *<0.001* | | *0.001* | | *0.002* | | *0.001* | | *0.028* | | *<0.001* | | *<0.001* | | *0.058* | | *0.018* | | *<0.001* | | *<0.001* | | *0.143* | | *<0.001* | | *<0.001* | | *0.177* | | *<0.001* | | *0.032* | | *0.003* | |
| **Subjective status** | |  | |  | |  | |  | |  | |  | |  | |  | |  | |  | |  | |  | |  | |  | |  | |  | |  | |  | |  | |  | |  | |  |
| Range 1-10 | 2.48  1.91-3.06 | | 1.33  -0.75-3.42 | | 1.80  1.10-2.50 | | 1.18  -0.11-2.48 | | 3.01  2.34-3.68 | | 4.22  2.45-5.99 | | 1.19  -0.24-2.62 | | 3.28  1.94-4.63 | | 2.78  1.32-4.24 | | 2.04  0.50-3.57 | | 1.65  0.49-2.82 | | 3.28  1.95-4.61 | | 2.13  0.54-3.73 | | 2.41  1.53-3.30 | | 2.50  1.82-3.19 | | 0.75  -0.66-2.15 | | -0.53  -1.75-0.70 | | 1.32  0.62-3.03 | | 3.07  1.70-4.45 | | 2.00  1.42-2.58 | | 1.70  0.31-3.08 | | 1.91  0.16-3.66 | |
| Linear trends | 2.46  1.88-3.03 | | 1.46  -0.58-3.50 | | 1.87  1.17-2.57 | | 1.15  -0.13-2.43 | | 2.97  2.30-3.64 | | 3.97  2.21-5.74 | | 1.25  -0.16-2.66 | | 3.24  1.92-4.56 | | 2.72  1.26-4.18 | | 2.65  1.05-4.24 | | 1.65  0.48-2.83 | | 3.16  1.84-4.49 | | 2.03  0.49-3.57 | | 2.40  1.51-3.28 | | 2.49  1.81-3.18 | | 0.72  -0.67-2.12 | | -0.65  -1.86-0.56 | | 1.33  0.62-2.03 | | 2.85  1.48-4.22 | | 2.02  1.44-2.59 | | 1.77  0.38-3.16 | | 1.86  0.11-3.62 | |
| *p-value* | *<0.001* | | *0.209* | | *<0.001* | | *0.078* | | *<0.001* | | *<0.001* | | *0.101* | | *<0.001* | | *<0.001* | | *0.010* | | *0.006* | | *<0.001* | | *0.009* | | *<0.001* | | *<0.001* | | *0.297* | | *0.398* | | *<0.001* | | *<0.001* | | *<0.001* | | *0.016* | | *0.032* | |

*Abbreviations:* MHI-5: Coeff: Coefficients; CI: Confidence interval; 5-item Mental Health Index. Ref: Reference group. Results based on imputed data. Models adjusted for age, gender, employment status, SCI severity, etiology, time since injury, mobility classes, and all SES indicators in the same model. Linear trends for financial hardship indicate lower mental health with increasing severity of financial hardship. Results supporting the expected direction of association with p<0.05 are marked in green, Results supporting the expected direction of association but with p>0.05 are marked in light green.
