# Supplementary material for: Socioeconomic Status, the Countries’ Socioeconomic Development and Mental Health: Observational Evidence for Persons with Spinal Cord Injury from 22 Countries
Source: Int J Public Health. 2022 Nov 30;67:1604673. doi: 10.3389/ijph.2022.1604673 (PMC9747630; doi:10.3389/ijph.2022.1604673)
Supplement: Supplementary file 2 [file DataSheet2.DOCX]

**Supplementary Table 2.** Unadjusted association of socioeconomic status and mental health: β-coefficients and 95% confidence intervals from linear regressions, stratified by the 22 countries participating in the International Spinal Cord Injury community survey (22 countries, 2017-2019).

|  | **Mental health**  (MHI-5 score 0-100, higher scores=better mental health) | | | | | | | | | | | | | | | | | | | | | |
| --- | --- | --- | --- | --- | --- | --- | --- | --- | --- | --- | --- | --- | --- | --- | --- | --- | --- | --- | --- | --- | --- | --- |
|  | Australia (n=1579) | Brazil (n=201) | China (n=1354) | France (n=412) | Germany (n=1617) | Greece (n=200) | Indonesia (n=201) | Italy  (n=206) | Japan (n=302) | Lithuania (n=218) | Malaysia  (n=297) | Morocco (n=385) | Netherlands (n=260) | Norway (n=609) | Poland (n=971) | Romania  (n=216) | South Africa (n=200) | South Korea (n=890) | Spain (n=417) | Switzerland (n=1530) | Thailand (n=320) | United States (n=203) |
|  | Coeff  95% CI | Coeff  95% CI | Coeff  95% CI | Coeff  95% CI | Coeff  95% CI | Coeff  95% CI | Coeff  95% CI | Coeff  95% CI | Coeff  95% CI | Coeff  95% CI | Coeff  95% CI | Coeff  95% CI | Coeff  95% CI | Coeff  95% CI | Coeff  95% CI | Coeff  95% CI | Coeff  95% CI | Coeff  95% CI | Coeff  95% CI | Coeff  95% CI | Coeff 95% CI | Coeff 95% CI |
| **Education** |  |  |  |  |  |  |  |  |  |  |  |  |  |  |  |  |  |  |  |  |  |  |
| No, primary, or lower secondary | Ref | Ref | Ref | Ref | Ref | Ref | Ref | Ref | Ref | Ref | Ref | Ref | Ref | Ref | Ref | Ref | Ref | Ref | Ref | Ref | Ref | Ref |
| Higher or post-secondary | 2.55  -0.11-5.20 | -0.72  -8.09-6.66 | -0.28  -2.51-1.96 | 4.49  -1.59-8.58 | 6.94  2.73-11.14 | 11.85  2.76-20.93 | -0.01  -5.66-5.64 | -2.55  -11.40-6.30 | 3.99  -2.31-10.29 | 10.17  2.94-17.40 | -1.82  -8.55-4.90 | 3.25  -1.81-8.32 | 1.77  -4.60-8.15 | 3.50  -0.97-7.97 | 3.77  0.97-6.56 | -5.21  -24.71-14.30 | 9.90  3.24-16.55 | 3.79  -0.26-7.84 | 6.78  0.66-12.90 | 4.22  1.05-7.40 | -1.13  -5.96-3.70 | 12.03  -23.20-47.25 |
| Tertiary | 5.18  2.58-7.78 | -0.60  -8.75-7.54 | 6.07  1.95-10.19 | 4.64  -0.68-9.96 | 9.59  5.02-14.17 | 16.04  5.22-26.87 | -0.82  -10.50-8.86 | 5.65  -5.47-16.77 | 7.74  0.51-14.98 | 4.33  -1.42-10.07 | 1.07  -6.19-8.33 | 2.01  -4.75-8.77 | 3.45  -2.09-8.99 | 6.42  2.01-10.82 | 7.67  4.41-10.93 | 1.55  -17.65-20.76 | 17.57  9.63-25.51 | 5.29  1.16-9.42 | 10.69  5.10-16.27 | 6.36  3.03-9.69 | -2.85  -8.48-2.77 | 11.84  -22.90-46.58 |
| *p-value* | *0.001* | *0.980* | *0.006* | *0.205* | *<0.001* | *0.010* | *0.981* | *0.126* | *0.107* | *0.022* | *0.454* | *0.445* | *0.467* | *0.010* | *<0.001* | *0.062* | *<0.001* | *0.043* | *0.001* | *0.001* | *0.609* | *0.771* |
| **Household income** |  |  |  |  |  |  |  |  |  |  |  |  |  |  |  |  |  |  |  |  |  |  |
| Lowest quartile | Ref | Ref | Ref | Ref | Ref | Ref | Ref | Ref | Ref | Ref | Ref | Ref | Ref | Ref | Ref | Ref | Ref | Ref | Ref | Ref | Ref | Ref |
| 2^nd^ lowest quartile | 4.53  1.40-7.66 | 1.61  -6.92-10.14 | 6.05  3.34-8.77 | 0.75  -4.49-5.99 | 3.93  0.83-7.04 | -5.40  -15.20-4.39 | 5.24  -2.09-12.58 | 2.05  -5.97-10.06 | -2.30  -8.57-3.97 | 8.25  0.98-15.52 | -1.05  -6.86-4.77 | -2.48  -9.11-4.15 | 6.53  -0.81-13.86 | 2.98  -1.24-7.20 | 1.15  -2.40-4.71 | -0.65  -7.94-6.63 | -2.94  -10.19-4.31 | 4.85  0.62-9.08 | -0.48  -6.98-6.01 | 1.15  -1.83-4.13 | 2.89  -3.27-9.05 | 1.12  -6.41-8.65 |
| 2^nd^ highest quartile | 5.06  1.92-8.20 | 0.18  -9.14-9.49 | 13.10  10.33-15.86 | 0.87  -4.76-6.51 | 6.07  2.97-9.17 | 6.42  -3.19-16.03 | 5.13  -2.23-12.49 | -0.41  -7.91-7.09 | 4.04  -2.81-10.89 | 5.00  -2.34-12.35 | -2.40  -8.20-3.40 | 3.82  -2.99-10.63 | 4.10  -2.99-11.20 | 4.80  0.34-9.25 | 5.29  1.75-8.82 | 6.96  -0.33-14.24 | 4.44  -3.93-12.80 | 4.50  0.70-8.31 | 10.35  4.07-16.62 | 4.16  1.28-7.05 | 2.89  -3.63-9.42 | 2.16  -4.75-9.07 |
| Highest quartile | 10.09  6.90-13.29 | 0.84  -8.52-10.19 | 15.13  12.42-17.83 | 6.82  1.50-12.14 | 11.36  8.33-14.39 | 5.68  -4.20-15.55 | -0.32  -7.83-7.20 | -5.23  -13.02-2.56 | 7.25  0.52-13.99 | 2.30  -4.90-9.50 | -0.20  -6.06-5.66 | 1.62  -5.13-8.37 | 6.59  -0.62-13.80 | 9.54  5.19-13.89 | 7.52  3.89-11.16 | 4.82  -2.55-12.18 | 18.91  11.52-26.29 | 7.57  3.82-11.31 | 12.07  5.72-18.41 | 6.15  3.31-8.98 | 5.52  -0.72-11.76 | 7.51  -1.74-16.75 |
| *p-value* | *<0.001* | *0.982* | *<0.001* | *0.048* | *<0.001* | *0.069* | *0.243* | *0.305* | *0.023* | *0.144* | *0.847* | *0.278* | *0.235* | *<0.001* | *<0.001* | *0.121* | *<0.001* | *<0.001* | *<0.001* | *<0.001* | *0.397* | *0.432* |
| **Financial hardship** |  |  |  |  |  |  |  |  |  |  |  |  |  |  |  |  |  |  |  |  |  |  |
| Massive | Ref | Ref | Ref | Ref | Ref | Ref | Ref | Ref | Ref | Ref | Ref | Ref | Ref | Ref | Ref | Ref | Ref | Ref | Ref | Ref | Ref | Ref |
| Some | 12.19  8.79-15.45 | 4.73  -3.38-12.83 | 9.01  6.48-11.53 | 5.07  -2.02-12.16 | 8.93  4.62-13.25 | 15.23  4.95-25.51 | 6.42  0.49-12.34 | 11.74  4.05-19.43 | 10.82  0.97-20.67 | 14.33  724-21.43 | 9.34  3.86-14.82 | 7.42  2.26-12.58 | 11.70  0.37-23.02 | 9.64  3.52-15.75 | 11.27  8.11-14.43 | 1.75  -5.13-8.64 | 4.58  -1.67-10.83 | 12.19  8.82-15.57 | 0.38  -7.40-8.17 | 6.47  2.57-10.37 | 3.07  -2.36-8.50 | 5.63  -4.04-15.31 |
| None | 19.30  16.41-22.19 | 3.91  -3.58-11.39 | 15.98  13.42-18.54 | 11.67  5.28-18.06 | 17.76  13.94-21.57 | 22.04  12.39-31.69 | 14.26  7.57-20.95 | 22.82  15.52-30.13 | 16.52  7.69-25.35 | 14.02  6.80-21.25 | 11.66  6.24-17.07 | 18.67  9.73-27.61 | 18.30  8.45-28.15 | 19.86  14.60-25.13 | 19.01  16.04-21.99 | 8.12  1.43-14.82 | 21.04  13.81-28.26 | 17.55  13.87-21.23 | 10.48  3.75-17.22 | 15.68  12.33-19.04 | 10.95  5.62-16.28 | 16.50  7.56-25.44 |
| *p-value* | *<0.001* | *0.457* | *<0.001* | *<0.001* | *<0.001* | *<0.001* | *<0.001* | *<0.001* | *<0.001* | *<0.001* | *<0.001* | *<0.001* | *<0.001* | *<0.001* | *<0.001* | *0.033* | *<0.001* | *<0.001* | *<0.001* | *<0.001* | *<0.001* | *<0.001* |
| **Subjective social status** | |  |  |  |  |  |  |  |  |  |  |  |  |  |  |  |  |  |  |  |  |  |
| Range 1-10 | 3.42  2.95-3.88 | 0.87  -0.82-2.57 | 3.51  2.96-4.06 | 2.08  1.05-3.11 | 3.93  3.37-4.84 | 4.84  3.20-6.49 | 1.61  0.40-2.81 | 4.72  3.53-5.91 | 3.82  2.59-5.05 | 2.09  0.68-3.50 | 1.64  0.56-2.72 | 3.49  2.36-4.63 | 2.69  1.55-3.84 | 3.33  2.66-4.01 | 3.69  3.12-4.26 | 1.63  0.26-3.00 | 0.52  -0.68-1.72 | 2.01  1.36-2.65 | 4.33  3.16-5.50 | 2.76  2.29-3.23 | 2.08  0.92-3.23 | 2.81  1.48-4.14 |
| *p-value* | *<0.001* | *0.312* | *<0.001* | *<0.001* | *<0.001* | *<0.001* | *<0.009* | *<0.001* | *<0.001* | *0.004* | *0.003* | *<0.001* | *<0.001* | *<0.001* | *<0.001* | *0.020* | *0.391* | *<0.001* | *<0.001* | *<0.001* | *<0.001* | *<0.001* |

*Abbreviations:* Coeff: Coefficient; CI: Confidence interval; MHI-5: 5-item Mental Health Index. Ref: Reference group. Results based on imputed data. Note that education was categorized differently in China, Indonesia, Morocco and Thailand (Reference = no schooling or primary; lower, higher or post-secondary; tertiary); *Color coding:* Results supporting the expected direction of association with p<0.05 are marked in green, Results supporting the expected direction of association but with p>0.05 are marked in light green.
