# Supplementary material for: Socioeconomic Status, the Countries’ Socioeconomic Development and Mental Health: Observational Evidence for Persons with Spinal Cord Injury from 22 Countries
Source: Int J Public Health. 2022 Nov 30;67:1604673. doi: 10.3389/ijph.2022.1604673 (PMC9747630; doi:10.3389/ijph.2022.1604673)
Supplement: Supplementary file 3 [file DataSheet1.DOCX]

**Electronic Supplementary Material**

**Supplementary Table 1.** Indicators of socioeconomic status in the 12,588 participants of the International Spinal Cord Injury community survey (22 countries, 2017-2019).

|  |  |  | **Highest level of education** ^a^ | | |  | **Net-equivalence household income** | | **Financial hardship** | | | | **Subjective social status** | |
| --- | --- | --- | --- | --- | --- | --- | --- | --- | --- | --- | --- | --- | --- | --- |
|  |  | **Total** | None, primary, or lower secondary | Higher or post-secondary | Tertiary |  |  |  | None | Some | Massive |  | Score 1-10 |  |
| Country |  | N | N (%) | N (%) | N (%) | *m* | Monthly mean (SD) in local currency | *m* | N (%) | N (%) | N (%) | *m* | Mean (SD) | *m* |
| Australia | AUS | 1,579 | 497 (32.0) | 499 (32.2) | 555 (35.8) | 1.8 | 3122.7 (2750.6) AUD ^b^ | 12.8 | 908 (59.7) | 372 (24.5) | 241 (15.8) | 3.7 | 5.0 (2.2) | 4.2 |
| Brazil | BRA | 201 | 67 (33.3) | 80 (39.8) | 54 (26.9) | 0 | 2084.7 (2144.3) BRL ^d^ | 1.0 | 80 (40.0) | 58 (29.0) | 62 (31.0) | 0.5 | 4.7 (1.9) | 1.5 |
| China | CHN | 1,354 | 391 (28.8) ^a^ | 864 (63.8) ^a^ | 99 (7.3) | 0 | Jiangsu: 2400.0 (2323.6) ¥ ^c,e^; Sichuan: 1458.5 (1496.0) ¥ ^c,e^ | 0 | 512 (37.8) | 551 (40.7) | 291 (21.5) | 0 | 3.7 (1.7) | 0 |
| France | FRA | 413 | 89 (22.0) | 173 (42.8) | 142 (35.2) | 1.9 | 1594.4 (704.9) € ^d^ | 7.3 | 260 (66.7) | 94 (24.1) | 36 (9.2) | 5.3 | 5.2 (1.8) | 3.9 |
| Germany | GER | 1,617 | 119 (7.7) | 1,092 (71.1) | 326 (21.2) | 5.0 | 1572.3 (903.6) € ^c^ | 12.3 | 1,068 (73.4) | 268 (18.4) | 120 (8.2) | 10.0 | 5.3 (1.9) | 6.6 |
| Greece | GRE | 200 | 38 (19.0) | 119 (59.5) | 43 (21.5) | 0 | 1160.1 (943.9) € ^b^ | 1.5 | 95 (50.3) | 62 (32.8) | 32 (16.9) | 5.5 | 5.3 (2.0) | 2.0 |
| Indonesia | INA | 201 | 63 (32.0) ^a^ | 117 (59.4) ^a^ | 17 (8.6) | 2.0 | 1,014,000.9 (1,572,000.1) Rp ^c^ | 3.0 | 51 (26.3) | 89 (45.9) | 54 (27.8) | 3.5 | 4.2 (2.1) | 2.5 |
| Italy | ITA | 206 | 20 (9.9) | 157 (77.7) | 25 (12.4) | 1.9 | 1393.1 (806.6) € ^d^ | 3.9 | 84 (43.1) | 78 (40.0) | 33 (16.9) | 5.3 | 5.2 (2.0) | 6.8 |
| Japan | JPN | 302 | 53 (17.6) | 175 (58.1) | 73 (24.3) | 0.3 | 180,000.5 (120,000.4) Yen ^c^ | 7.6 | 214 (74.1) | 53 (18.3) | 22 (7.6) | 4.3 | 4.7 (1.8) | 10.6 |
| Lithuania | LTU | 218 | 75 (34.7) | 42 (19.4) | 99 (45.8) | 0.9 | 470.1 (256.3) € ^c^ | 0.9 | 83 (38.4) | 95 (44.0) | 38 (17.6) | 0.9 | 5.5 (1.8) | 0.5 |
| Malaysia | MAS | 298 | 34 (11.5) | 171 (57.8) | 91 (30.7) | 0.3 | 1195.0 (1369.5) RM ^b^ | 2.7 | 118 (41.8) | 108 (38.3) | 56 (19.9) | 5.1 | 4.4 (1.9) | 2.0 |
| Morocco | MAR | 385 | 151 (39.2) ^a^ | 170 (44.2) ^a^ | 64 (16.6) | 0 | 1098.9 (1045.7) DH ^d^ | 0 | 27 (7.0) | 103 (26.8) | 255 (66.2) | 0 | 3.3 (1.9) | 0 |
| Netherlands | NED | 260 | 99 (38.7) | 57 (22.3) | 100 (39.1) | 1.5 | 2041.0 (936.0) € ^d^ | 11.2 | 196 (78.1) | 39 (15.5) | 16 (6.4) | 3.5 | 6.0 (2.0) | 6.9 |
| Norway | NOR | 610 | 92 (15.3) | 239 (39.8) | 270 (44.9) | 1.3 | 420,252.1 (201,844.4) NOK ^b^ | 4.9 | 448 (76.1) | 98 (16.6) | 43 (7.3) | 3.3 | 5.7 (2.0) | 3.9 |
| Poland | POL | 971 | 422 (43.6) | 342 (35.3) | 205 (21.2) | 0.2 | 1,556.0 (1,110.6) ZŁ ^c^ | 5.3 | 415 (44.4) | 302 (32.3) | 217 (23.2) | 3.8 | 4.6 (2.0) | 1.7 |
| Romania | ROU | 216 | 4 (1.9) | 68 (31.5) | 144 (66.7) | 0 | 1,319.5 (855.6) RON ^c^ | 1.4 | 82 (39.4) | 72 (34.6) | 54 (26.0) | 3.7 | 4.8 (1.9) | 2.3 |
| South Africa | RSA | 200 | 58 (29.2) | 96 (48.2) | 45 (22.6) | 0.5 | 6,677.0 (11,203.5) R ^c^ | 1.5 | 42 (21.1) | 67 (33.7) | 90 (45.2) | 0.5 | 4.4 (2.5) | 0.5 |
| South Korea | KOR | 890 | 152 (17.2) | 390 (44.2) | 340 (38.6) | 0.9 | 1,350,000.3 (1,220,000.8) ₩ ^c^ | 4.4 | 252 (28.9) | 392 (45.0) | 228 (26.2) | 2.0 | 4.1 (2.2) | 8.0 |
| Spain | ESP | 417 | 113 (27.2) | 122 (29.3) | 181 (43.5) | 0.2 | 1,241.1 (801.4) € ^c^ | 6.7 | 248 (62.6) | 91 (23.0) | 57 (14.4) | 5.0 | 5.0 (1.9) | 4.1 |
| Switzerland | SUI | 1,530 | 151 (10.3) | 771 (52.6) | 543 (37.1) | 4.3 | 4,508.1 (2,034.8) CHF ^b^ | 24.0 | 1,092 (74.5) | 252 (17.2) | 121 (8.3) | 4.3 | 5.6 (2.0) | 6.7 |
| Thailand | THA | 320 | 111 (34.8) ^a^ | 134 (42.0) ^a^ | 74 (23.2) | 0.3 | 9,781.6 (11,975.2) ฿ ^d^ | 2.8 | 127 (40.1) | 116 (36.6) | 74 (23.3) | 3 | 4.0 (1.8) | 3.4 |
| United States | USA | 203 | 1 (0.5) | 59 (29.8) | 138 (69.7) | 2.5 | 7,984.2 (5,406.5) $ ^b^ | 8.9 | 121 (63.4) | 54 (28.3) | 16 (8.4) | 5.9 | 6.0 (1.9) | 3.9 |

Results based on full cases, m= % of missing values in the country sample.

^a^ Different coding scheme for China, Indonesia, Morocco and Thailand: first educational group includes persons without schooling or primary education; Second group: lower, upper and post-secondary, third group: tertiary.

^b^ Household income before taxes, ^c^ Household income after taxes; ^d^ Item did not specify whether to indicate income before/after taxes. ^e^ Distribution-based quartiles were built separately for Jiangsu and Sichuan given the large differences in income levels between the two Provinces.
